# Supplementary material for: Latitudinal gradient in dairy production with the introduction of farming in Atlantic Europe
Source: Nat Commun. 2020 Apr 27;11:2036. doi: 10.1038/s41467-020-15907-4 (PMC7184739; doi:10.1038/s41467-020-15907-4)
Supplement: Supplementary file 1 — Supplementary Information [file 41467_2020_15907_MOESM1_ESM.pdf]

Supplementary Information for

Latitudinal gradient in dairy production with the introduction of farming in Atlantic Europe

Cubas et al.

Contents:

Supplementary Note 1: Additional archaeological information

Supplementary Tables 1-4

Supplementary References

Supplementary Note 1: Additional archaeological information

CENTRAL-SOUTHERN PORTUGAL

Six archaeological sites from Portugal were selected. The majority of them are located in the area of Lisbon and they correspond to different typologies of archaeological sites. We selected shell-middens (Cabeço das Amoreiras), caves (Gruta do Caldeirão) and open air sites (Lapiás das Lameiras, São Pedro de Canaferrim, Valado do Mato and Monte da Foz 1). The pottery assemblages recorded from two sites in Portugal (Cabeço das Amoreiras and Monte da Foz 1) are attributed to this period based on typology[1-3]. Cardial wares are present in Cabeço das Amoreiras[1], Gruta do Caldeirão[4], Monte da Foz I[3] and Valada do Mato[5] but completely absent in other Early Neolithic sites such as Lapiás das Lameiras and São de Pedro de Canaferrim[6].

NORTHERN SPAIN

Three archaeological sites from Spain were selected. They correspond with the earliest pottery assemblages in the north of Spain. Cardial ware pottery from Cova Eirós came from the bottom of a heavily disturbed pit, although its Early Neolithic assignation is undeniable[7]. This decorative pattern is absent in the archaeological sites of Los Canes and Los Gitanos.

FRANCE

Six archaeological sites from Atlantic France were selected. They represent open-air sites with domestic structures (Lannion “Kervouric”, Verson “Les Mesnils”, Colombelles “Le Lazzaro” and Démouville). In addition, two of the most paradigmatic sites with La Hoguette pottery from Normandy (Fontenay le Marmion and Alizay) were sampled. Two archaeological sites lacked radiocarbon dates but were selected based on the relevance of their pottery assemblages for understanding the Early Neolithic of this region. Fontenay-le-Marmion “La Hoguette” is a tumulus with the main occupation phase dated to the Middle Neolithic. However, in the lower part of the funerary chamber two pottery vessels were recorded[8]. One of these pots was selected for analysis. Démouville and Colombelles “Le Lazzaro” yielded the earliest evidence for Neolithic occupation in Normandy containing Rubané wares although some Limburg pottery is present in this latter assemblage[9]. Samples from Atlantic France can be attributed to the end of Rubané and the Blicquy-Villeneuve Saint Germain (Lannion “Kervouric”, Verson “Les Mesnils” and Colombelles)[9-11].

WESTERN BALTIC

Seven Early Neolithic Funnel Beakers (hereafter TRB) from the transitional settlement sites of Åkonge and Stenø, located in the Åmose on the island of Zealand, Denmark were selected. In addition, five TRB vessels from two areas (MLF906-1; MLF939-1) of the Syltholm excavations

(east of Rødby Harbour) on the island of Lolland were included. Furthermore, nine TRB vessels from four find spots (Jordløse Mose XX, Jordløse Mose XXI, Maglelyng 2, and Målevgård Mose) situated in the aforementioned Åmose and one TRB vessel from Neverkær Mose on the island of Fyn were sampled. Although three of the find spots lacked AMS radiocarbon (^{14}C) dates, they were confidently assigned as Funnel Beakers based on the typological analysis undertaken by Koch[12].

Supplementary Table 1. Summary of pottery samples analysed directly in this study. *Approximate chronology is based on available calibrated radiocarbon dates for each site when n o radiocarbon dates were available, most representative vessel typology is highlighted. **Estimat ed % of samples has been established considering the *n* number of vessels, when available, or the total *n* of pottery sherds recovering in the same archaeological context. ***Lipid concentration w as quantified from the acidified methanol extract only. "n.d." indicates that no data are available.

(next page)

Country/region	Site	Site type	Date of context (cal BC at 95%) *	*n* samples	Estimated % of samples**	Total *n* of sherds	Total *n* of vessels	*n* (>5 µg g^{-1} lipid concentration)***	% dairy fats
Central-Southern Portugal	Gruta do Caldeirão	Cave	5,500-4,500	8	53.3	n.d.	15	8	12.5
	Lapiás das Lameiras	Open air	5,500-5,000	9	n.d.	n.d.	n.d.	8	12.5
	São Pedro de Canaferrim	Open air	5,300-4,700	9	22.5	40	9	8	12.5
	Cabeço das Amoreiras	Open air	n.d. Cardial	15	n.d.	n.d.	n.d.	15	0
	Valada do Mato	Open air	5,500-4,500	21	7.8	1,240	268	21	0
	Monte da Foz	Open air	n.d. Cardial	16	9.8	2,563	163	16	6.3
Northern Spain	Los Canes	Cave	5,000-4,500	8	10.4	77	n.d.	8	25
	Los Gitanos A4	Cave	4,500-3,500	14	36.8	38	n.d.	13	23.1
	Los Gitanos A3	Cave	5,000-3,500	14	45.2	31	n.d.	14	21.4

	Cova Eirós	Cave	n.d. Cardial	1	100	1	1	1	0
France	Alizay	Open air	5,400-5,200	2[1]	100	1	1	2	100
	Fontenay-le-Marmion	Megalithic structure	n.d. La Hoguette	1	100	1	1	1	0
	Lannion “Kervouric”	Open air	4,900-4,600	22	100	616	22	19	42.1
	Colombelles “Le Lazzaro”	Open air	5,300-4,900	39	44.8	n.d.	87	39	43.6
	Verson “Les Mesnils”	Open air	5,200-4,000	42	9.1	8040	464	36	44.4
	Démouville	Open air	n.d.	3	n.d.	n.d.	n.d.	3	33.3
Western Baltic	Åkonge	Waterlogged lakeshore	3,950-3,650	1	n.d.	1	1	1	100
	Jordløse Mose XX	Waterlogged find spot	3,950–3,530	4	66.7	6	3	4	25
	Jordløse Mose XXI	Waterlogged find spot	n.d. Funnel Beaker	1	100	1	1	1	100

[1] Two sherds (rim and bottom) from the same vessel were analysed.

	Maglelyng 2	Waterlogged find spot	n.d. Funnel Beaker	2	100	2	2	2	50
	Målevgård Mose	Waterlogged find spot	3,950–3,650	2	100	2	2	2	100
	Neverkær	Waterlogged find spot	n.d. Funnel Beaker	1	100	1	1	1	0
	Rødbyhavn (MLF906-1)	Waterlogged coastal	3,950-3,300	4	n.d.	n.d.	n.d.	4	50
	Rødbyhavn (MLF939-1)	Waterlogged coastal	3,950-3,300	1	n.d.	n.d.	n.d.	1	100
	Stenø	Waterlogged lakeshore	3,950	6	n.d.	n.d.	n.d.	6	50

Supplementary Table 2. Summary of $\delta^{13}C$ values of $C_{16:0}$ and $C_{18:0}$ *n*-alkanoic acids obtained from modern European authentic reference fats and oils (ruminant, non-ruminant, dairy and marine)[13-20] corrected for the Suess effect. These were used to create reference elipses in Fig. 2 and for the Bayesian mixing model. Concentration of $C_{16:0}$ and $C_{18:0}$ *n*-alkanoic acids in the different products obtained from the USDA Food Composition Databases (https://ndb.nal.usda.gov/ndb/) are also summarised.

**FOOD sources**	**Source values (‰)**			**Concentrations (% of total lipid)**	
	$\delta^{13}C_{16:0}$	$\delta^{13}C_{18:0}$	$\Delta^{13}C(C_{18:0}-C_{16:0})$	$C_{16:0}$	$C_{18:0}$
**Porcine adipose fats**					
Mean	-25.2	-24.4	0.8	20.5%	10.9%
Standard deviation	0.8	0.9	0.6	2.5%	1.7%
N	66	66	66	336	
**Ruminant adipose fats**					
Mean	-29.1	-30.7	-1.6	21.1%	13.4%
Standard deviation	1.3	1.9	1.0	3.1%	3.0%
N	32	32	66	1345	
**Ruminant dairy fats**					
Mean	-28.7	-33.6	-4.8	27.3%	11.0%
Standard deviation	1.7	2.4	1.4	3.5%	1.7%
N	36	36	66	120	
**Marine oils**					
Mean	-22.8	-22.3	0.5	14.5%	4.0%
Standard deviation	2.6	2.6	1.2	4.3%	2.2%
N	100	100	66	231	

Supplementary Table 3. Median stable carbon isotope ($\delta^{13}C$) values of $C_{16:0}$ and $C_{18:0}$ *n*-alkanoic acids from each region used in the Bayesian semiparametric mixed model. The model was applied to the median isotope values from each region, excluding the western Baltic where the data are bimodally distributed between marine and terrestrial values. These values are based on isotopic values provided in Supplementary Data 2.

Location	$\delta^{13}C_{16:0}$			$\delta^{13}C_{18:0}$			*n*
	Mean	Std. dev.	Median	Mean	Std. dev.	Median	
France & Channel Islands	-29.5	1.1	-29.7	-32.3	1.9	-32.7	107
North England, Scotland and Ireland	-28.1	1.0	-28.1	-32.9	1.4	-33.1	187
Central-Southern Portugal	-27.6	1.4	-27.6	-28.6	1.3	-28.8	68
South England	-27.9	1.0	-27.8	-31.4	2.2	-31.9	165
Northern Spain	-28.4	1.0	-28.3	-30.2	1.9	-29.8	36

Supplementary Table 4. Faunal remains from assemblages recovered from the sampled sites. The Number of Identified Specimens (NISP) values are reported. "n.d." indicates that data are not yet available/absent, whilst “p.” indicates presence without quantification". Sites with fish or/and shellfish remains are highlighted in bold.

(next page)

Country/region	Archaeological site	UE	Wild ruminant	Wild non-ruminant	Non-ruminant (non identified)	Domestic ruminant				**Reference**
						**Cattle**	**Sheep/goat**	**Sheep**	**Goat**	
Central-Southern Portugal	Gruta do Caldeirão	NA1	15	78	---	20	14	5	---	21
		NA2	1	18	---	---	---	1	---	21
	**Lameiras**	---	5	---	117.5	15	48	44.5	14	22
	São Pedro de Canaferrim	No preservation								22
	**Cabeço das Amoreiras**	---	n.d.	n.d.	n.d.	n.d.	n.d.	n.d.	n.d.	n.d
	**Arapouco**	---	n.d.	n.d.	n.d.	n.d.	n.d.	n.d.	n.d.	n.d
	Valada do Mato	---	---	---	1		2	---	---	5
	Monte da Foz	No preservation								3
Northern Spain	Los Canes	7	33	---	---	---	---	---	---	23
	**Los Gitanos**	A4	80	10	14	1	40	9	---	24
		A3	16	---	11	2	18	3	---	24
	Cova Eirós	---	---	---	---	---	---	---	---	7
France	Alizay	---	---	---	---	---	---	---	---	8
	Fontenay-le-Marmion	---	---	---	---	---	---	---	---	8
	Lannion “Kervouric”	No preservation								10
	Colombelles “Le Lazzaro”	126	0	---	---	---	2	---	---	9
		22	0	1	---	---	3	---	---	9
		379	0	---	---	---	4	---	---	9

		380	0	---	---	---	---	---	---	9
		464	0	---	---	---	---	---	---	9
	Verson “Les Mesnils”	No preservation								11
	Demouville	n.d.	n.d.	n.d.	n.d.	n.d.	n.d.	n.d.	n.d.	n.d.
Western Baltic	**Åkonge**	---	2125	189	---	16	---	---	---	25,26,27
	Jordløse Mose XX	---	n.d.	n.d.	n.d.	n.d.	n.d.	n.d.	n.d.	n.d
	Jordløse Mose XXI	---	n.d.	n.d.	n.d.	n.d.	n.d.	n.d.	n.d.	n.d
	Maglelyng 2	---	n.d.	n.d.	p.	n.d.	n.d.	n.d.	n.d.	n.d
	Målevgård Mose	---	n.d.	n.d.	n.d.	n.d.	n.d.	n.d.	n.d.	n.d
	Neverkær	---	n.d.	n.d.	n.d.	n.d.	n.d.	n.d.	n.d.	n.d
	Rødbyhavn (MLF906-1)	---	p.	p.	n.d.	n.d.	n.d.	n.d.	n.d.	n.d
	Rødbyhavn (MLF939-1)	---	p.	p.	n.d.	n.d.	n.d.	n.d.	n.d.	n.d
	Stenø	---	n.d.	n.d.	n.d.	n.d.	n.d.	n.d.	n.d.	n.d

Supplementary References

1. Arnaud, J. M. Os concheiros mesolíticos do vale do Sado e a explotaçao dos recursos estuarinos (nos tempos pré-históricos e na actualidade). *Trabalhos de Arqueologia* **14**, 21-43 (2000).
2. Diniz, D. in *Os últimos caçadores-recolectores e as primeiras comunidades produtoras do sul da Península Ibérica e do norte de Marrocos* (eds J. B. Gibaja Bao & A. F. Carvalho) 49-62 (Universidade do Algarve, Promontoria Arqueologica 15, 2010).
3. Neves, C. *Monte da Foz 1 (Benavente): um episódio da Neolitizaçao na margen esquerda do Baixo Tejo* (Unpublished Master Dissertation, Universidade de Lisboa, 2010).
4. Zilhão, J. *Gruta do Caldeirão. O Neolítico Antigo* (Instituto Português do Património Arquitectónico e Arqueológico. Trabalhos de Arqueología 6, 1992).
5. Diniz, D. *O Sítio da Valada do Mato (Évora): aspectos da neolitizaçao no Interior/Sul de Portugal* (Instituto Português de Arqueologia, Trabalhos de Arqueologia 48, 2007).
6. Simões, T. *O sítio neolítico de São Pedro de Canaferrim. Sintra* (Instituto Português de Arqueologia, Trabalhos de Arqueologia 12, 1999).
7. Fábregas Valcarce, R. *et al.* Vaso con decoración cardial de Cova Eirós. *Trabajos de Prehistoria* **76**, 147-160 (2019).
8. Ghesquiére, E. & Aubry, B. in *Transitions, ruptures et continuité en Préhistoire* 503-522 (Société Préhistorique Française 2013).
9. Billard, C., Bostyn, F., Hamon, C. & Meunier, K. *L'habitat du Néolithique ancien de Colombelles "Le Lazzaro" (Calvados)* (Société Préhistorique Française, 2014).
10. Juhel, L. *Un habitat du Néolithique ancien* (Unpublished Rapport final d'opération, 2015).
11. Germain-Vallée, C. *Un hameau du Néolithique ancien et les indices d'une occupation du Néolithique Moyen II* (Unpublished Rapport final d’opération, 2015).
12. Koch, E. *Neolithic Bog pots from Zealand, Møn, Lolland and Falster* (Det Kongelige Nordiske Oldskriftselskab, 1998).
13. Craig, O. E. *et al.* Ancient lipids reveal continuity in culinary practices across the transition to agriculture in Northern Europe. *Proc. Natl. Acad. Sci. USA* **108**, 17910-17915 (2011).
14. Cramp, L. J. E. *et al.* Immediate replacement of fishing with dairying by the earliest farmers of the northeast Atlantic archipelagos. *Proc. R. Soc. B* **281**, 20132372 (2014).
15. Dudd, S. N. *Molecular and isotopic characterization of animal fats in archaeological pottery* (Unpublished PhD, University of Bristol, 1999).

16. Spangenberg, J. E., Jacomet, S. & Schibler, J. r. Chemical analyses of organic residues in archaeological pottery from Arbon Bleiche 3, Switzerland: evidence for dairying in the late Neolithic. *J. Archaeol. Sci.* **33**, 1-13 (2006).

17. Bell, J. G. *et al.* Discrimination of Wild and Cultured European Sea Bass (Dicentrarchus labrax) Using Chemical and Isotopic Analyses. *J. Agric. Food Chem.* **55**, 5934-5941 (2007).

18. Spiteri, C. D. *Pottery use at the transition to agriculture in the western Mediterranean. Evidence from biomolecular and isotopic characterisation of organic residues in Impressed/Cardial ware vessels* (Unpublished PhD, University of York, 2012).

19. Recio, C., Martín, Q. & Raposo, C. GC-C-IRMS analysis of FAMEs as a tool to ascertain the diet of Iberian pigs used for the production of pork products with high added value. *Grasas y Aceites* **64**, 181-190 (2013).

20. Carrer, F. *et al.* Chemical Analysis of Pottery Demonstrates Prehistoric Origin for High-Altitude Alpine Dairying. *PLOS One* **11**, e0151442, doi: doi.org/10.1371/journal.pone.0151442 (2016).

21. Rowley-Conwy, P. in *Gruta do Caldeirão. O Neolítico Antigo.* (ed J. Zilhão) 231-257 (Instituto Português do Património Arquitectónico e Arqueológico, Trabalhos de Arqueología, 61992).

22. Davis, S. J. M., Gabriel, S. & Simões, T. Animal remains from Neolithic Lameiras, Sintra: the earliest domesticated sheep, goat, cattle and pigs in Portugal and some notes on their evolution. *Archaeofauna* **27**, 93-172 (2018).

23. Arias, P. *La cueva de Los Canes (Asturias). Los últimos cazadores de la Península Ibérica ante la muerte*. (Unpublished, 2002).

24. Ontañón-Peredo, R. *et al.* in *Anciens peuplements littoraux et relations Homme/ Milieu sur les côtes de l'Europe atlantique* (eds M-Y. Daire *et al.*) 383-390 (Archaeopress BAR International Series 2570, 2013).

25. Enghoff, I. B. Fishing in Denmark during the Ertebølle period. *Int. J. Osteoarchaeol.* **4**: 65–96 (1994).

26. Enghoff, I. B. Regionality and Biotope Exploitation in Danish Ertebølle and Adjoining Periods. *Scientia Danica. Series B. Biologica*. Vol. 1. København: Det Kongelige Danske Videnskabernes Selskab (2011).

27. Gotfredsen, A. B. En rekonstruktion af palæomiljøet omkring thre senmesolitske bopladser i Store Åmose, Vestsjælland-baseret på pattedyr- og fugleknogler. *Geologisk Tidsskrift* **2**: 92–103 (1998).

28. Robson, H. *Evaluating the change of consumption and culinary practices at the transition to agriculture: a multi-disciplinary approach from a Danish kitchen midden* (Unpublished PhD, University of York, 2015).

29. Craig, O. E. *et al.* Molecular and isotopic demonstration of the processing of aquatic products in Northern European Prehistoric pottery. *Archaeometry* **49**, 135-152 (2007).

30. Smyth, J. & Evershed, R. P. Milking the megafauna: using organic residue analysis to understand early farming practice. *Environ. Archaeol* **21**, 214-229 (2017).

31. Copley, M. S. *et al.* Dairying in antiquity. III. Evidence from absorbed lipid residues dating to the British Neolithic. *J. Archaeol. Sci.* **32**, 523-546 (2005).

32. Manning, K., Colledge, S., Crema, E., Shenan, S. & Timpson, A. The cultural evolution of Neolithic Europe. EUROEVOL Dataset 1: sites, phases and radiocarbon data. *J. Open Archaeol. Data* **5**, doi: 10.5334/joad.40 (2016).

33. Martins, H. *et al.* Radiocarbon dating the beginning of the Neolithic in Iberia: new results, new problems. *J. Mediterr. Archaeol.* **28**, 105–131 (2015).

34. Bernabeu Aubán, J., Orozco, T., Díez Castillo, A., Gómez Puche, M. & Molina Hernández, F. J. Mas d'Is (Penàguila, Alicante): Aldeas y recintos monumentales del neolítico inicial en el Valle del Serpis. *Trab. Prehist.* **60**, 39–59 (2003).

35. García Borja, P., Aura Tortosa, J. E., Bernabeu Aubán, J. & Jordá-Pardo, J. F. Nuevas perspectivas sobre la neolitización en la Cueva de Nerja (Málaga, España): la cerámica de la Sala del Vestíbulo. *Zephyrus* **66**, 109–132 (2010).

36. Baldellou, V. La Cueva de Chaves (Bastarás-Casbas, Huesca). *Saguntum-Extra (Las primeras producciones cerámicas el VI milenio cal AC en la Península Ibérica)* **12**, 141–144 (2011).

37. Bernabeu Aubán, J., Molina Balaguer, L., Esquembre Bebia, M. A., Ortega, J. R. & Boronat Soler, J. D. La cerámica impresa mediterránea en el origen del Neolítico de la Península Ibérica? in *De Méditerranée et d'ailleurs...(Mélanges offerts à Jean Guilaine)* 83–95 (Centre de la Recherche sur la Préhistoire et la Protohistoire de la Méditerranée (Archives d'Écologie Préhistorique), 2009).

38. Isern, N., Zilhão, J., Fort, J. & Ammerman, A. J. Modeling the role of voyaging in the coastal spread of the Early Neolithic in the West Mediterranean. *Proc. Natl. Acad. Sci.* **114**, 897–902 (2017).

39. García Borja, P., Cortell Pérez, E., Pardo Gordó, S. & Pérez Jordà, G. Las cerámicas de la Cova de l'Or (Beniarrés, Alacant). Tipología y decoración de las colecciones del Museu d'Alcoi. *Recer. del Mus. d'Alcoi* **20**, 71–136 (2011).

40. Morales Hidalgo, J. I., Fontanals Torroja, M., Oms Arias, F. X. & Vergès Bosch, J. M. The chronology of the cardial Neolithic in the NE of the Iberian Peninsula. Dating, problematic and methodology. *Anthropologie* **114**, 427–444 (2010).

41. Rojo Guerra, M. A., Kunst, M., Garrido Pena, R. & García Martínez de Lagrán, I. La Neolitización de la Meseta Norte a la luz del C-14: análisis de 47 dataciones absolutas inéditas de dos yacimientos domésticos del Valle de Ambrona, Soria, España. *Arch. Prehist. Levantina* **XXVI**, 39–100 (2006).

42. Vergès, J. M. *et al.* Los niveles neolíticos de la cueva de El Mirador (Sierra de Atapuerca, Burgos): nuevos datos sobre la implantación y el desarrollo de la economía agropecuaria en la submeseta norte. *IV Congreso del Neolítico Peninsular* vol. I 418–428 (2008).

43. Martínez, G., Afonso, J. A., Cámara, J. A. & F., M. Contextualización cronológica y análisis tecnotipológico de los artefactos tallados del Neolítico antiguo de Los Castillejos (Montefrío, Granada. *Promontoria* **15**, 163–171 (2010).

44. Rojo Guerra, M. A., Kunst, M., Garrido Pena, R., García Martínez de Lagrán, Í. & Morán, G. *Paisajes de la memoria: asentamiento del Neolítico antiguo en el Valle de Ambrona (Soria, España)*. (Universidad de Valladolid, 2008).

45. Peña-Chocarro, L., Pérez Jordá, G., Morales Mateos, J. & Vera Rodríguez, J. C. ...Y llegaron los agricultores: agricultura y recolección en el occidente del Mediterráneo. *Menga* **04**, 15-34 (2013).

46. Cortés Sánchez, M. *et al.* The Mesolithic-Neolithic transition in southern Iberia. *Quat. Res.* **77**, 221–234 (2012).

47. Oms Arias, F. X. *et al.* Hàbitat en cova i espai pels ramats ca. 6200-6000 BP: la Cova Colomera (Prepirineu de Lleida) durant el Neolític Antic. *Saguntum (P.L.A.V.)* **45**, 25–38 (2013).

48. Rojo Guerra, M. A. *et al.* Pastores transhumantes del Neolítico Antiguo en un entorno de alta montaña: secuencia crono-cultural de la Cova de Els Trocs (San Feliú de Veri, Huesca). *Boletín del Semin. Estud. Arte y Arqueol.* **LXXIX**, 9–56 (2013).

49. Bosch i Lloret, A., Chinchilla Sánchez, J. & Tarrús i Galter, J. *El poblat lacustre neolític de La Draga. Excavacions de 1990 a 1998.* (Museu d’Arqueologia de Catalunya. Centre d’Arqueologia Subaquàtica de Catalunya (Monografies del CASC, 2), 2000).

50. Carvalho, A. F. Novos dados sobre dois temas da Pré-História do Sul de Portugal: o Mirense e o processo de neolitização. *Promontoria* **5**, 91–110 (2007).

51. Rojo Guerra, M. A. *et al. Zafrín. Un asentamiento del Neolítico Antiguo en las Islas Chafarinas (Norte de África, España)*. (Universidad de Valladolid (Studia Archaeologica 6), 2010).

52. Straus, L. & González Morales, M. El Mirón Cave and the 14 C Chronology of Cantabrian Spain. *Radiocarbon* **45**, 41–58 (2003).

53. Zapata Peña, L., Ibáñez Estévez, J. & González Urquijo, J. El yacimiento de la cueva de Kobaederra (Oma, Kortezubi, Bizkaia). Resultados preliminares de las campanas de excavación 1995-97. *Munibe. Ciencias Nat.* **49**, 51–63 (1997).

54. Zapata Peña, L., Milner, N. & Roselló Izquierdo, E. Pico Ramos cave shell midden: The Mesolithic-Neolithic transition by the Bay of Biscay. in *Shell middens in Atlantic Europe* (eds. Milner, N., Craig, O. E. & Bailey, G. N.) 150–157 (Oxbow Books, 2007).

55. Alday Ruiz, A. & Mujika Alustiza, J. A. Nuevos datos de cronología absoluta concerniente al Holoceno Medio en el área vasca. *El Mundo indígena (XXIV Congreso Nacional de Arqueología)* 95–106 (1999).

56. Reimer, P. J. *et al.* Intcal13 and marine13 radiocarbon age calibration curves 0 – 50,000 years cal BP. *Radiocarbon* **55**, 1869-1887 (2013).

57. Haslett, J. & Parnell, A. C. A simple monotone process with application to radiocarbon-dated depth chronologies. *Appl. Statist.* **57**, 399–418 (2008).
