# Supplementary material for: Latitudinal gradient in dairy production with the introduction of farming in Atlantic Europe
Source: Nat Commun. 2020 Apr 27;11:2036. doi: 10.1038/s41467-020-15907-4 (PMC7184739; doi:10.1038/s41467-020-15907-4)
Supplement: Supplementary file 4 — Description of Additional Supplementary Files [file 41467_2020_15907_MOESM4_ESM.pdf]

Description of Additional Supplementary Files

File Name: Supplementary Data 1
Description: Site name, region, sample code, sherd morphology, vessel decoration (FB: Funnel Beaker), lipid concentration and lipid classes present of samples analysed in this study (FA: saturated fatty acid; UFA: unsaturated fatty acid; BrFA: branched fatty acid; DA: dicarboxilic acid; Alk: alkane; TMTD: trimethyltridecanoic acid; Pri: Pristanic acid; Phy: Phytanic acid; APAA: ω-(o-alkylphenyl) alkanoic acid; Terp: Terpene; Ket: Ketone; Alc: Alcohol; Alkes: Alkenes; Chl: Cholesterol; Sit: sitosterol; MAG: monoacylglycerols; DAG: diacylglycerols; TAG: triacylglycerols; WE: wax ester; nd: no data).

File Name: Supplementary Data 2
Description: Stable carbon isotope (δ13C) values of C16:0 and C18:0 n-alkanoic acids obtained on Early Neolithic pottery from the archaeological sites located in the Atlantic coast of Europe and the Western Baltic (n = 647) grouped by region. This dataset has been used to elaborate Figs.1, 2 and 3 and to run the mixing model. References are provided in Supplementary Information File.

File Name: Supplementary Data 3
Description: Correspondance numbers in Fig. 1A and archaeological sites. Mean and standard deviation values of Δ13C by site. Supplementary data for Fig. 1C
